# Monitoring indicator genes to assess antimicrobial resistance contamination in phytoplankton and zooplankton communities from the English Channel and the North Sea

**DOI:** 10.3389/fmicb.2024.1313056

**Published:** 2024-02-08

**Authors:** Erwan Bourdonnais, Cédric Le Bris, Thomas Brauge, Graziella Midelet

**Affiliations:** ^1^ANSES, Laboratoire de Sécurité des Aliments, Unité Bactériologie et Parasitologie des Produits de la Pêche et de l'Aquaculture, Boulogne-sur-Mer, France; ^2^Univ. du Littoral Côte d'Opale, UMR 1158 BioEcoAgro, Institut Charles Viollette, Unité sous Contrat ANSES, INRAe, Univ. Artois, Univ. Lille, Univ. de Picardie Jules Verne, Univ. de Liège, Junia, Boulogne-sur-Mer, France

**Keywords:** antimicrobial resistance, indicator genes, phytoplankton, zooplankton, marine environment, environmental factors

## Abstract

Phytoplankton and zooplankton play a crucial role in marine ecosystems as the basis of the food webs but are also vulnerable to environmental pollutants. Among emerging pollutants, antimicrobial resistance (AMR) is a major public health problem encountered in all environmental compartments. However, the role of planktonic communities in its dissemination within the marine environment remains largely unexplored. In this study, we monitored four genes proposed as AMR indicators (*tetA, bla*_TEM_, *sul1*, and *intI1*) in phytoplankton and zooplankton samples collected in the English Channel and the North Sea. The indicator gene abundance was mapped to identify the potential sources of contamination. Correlation was assessed with environmental parameters to explore the potential factors influencing the abundance of AMR in the plankton samples. The prevalence in phytoplankton and zooplankton of *sul1* and *intI1*, the most quantified indicator genes, ranged from 63 to 88%. A higher level of phytoplankton and zooplankton carrying these genes was observed near the French and English coasts in areas subjected to anthropogenic discharges from the lands but also far from the coasts. Correlation analysis demonstrated that water temperature, pH, dissolved oxygen and turbidity were correlated to the abundance of indicator genes associated with phytoplankton and zooplankton samples. In conclusion, the *sul1* and *intI1* genes would be suitable indicators for monitoring AMR contamination of the marine environment, either in phytoplankton and zooplankton communities or in seawater. This study fills a part of the gaps in knowledge about the AMR transport by marine phytoplankton and zooplankton, which may play a role in the transmission of resistance to humans through the marine food webs.

## Graphical Abstract

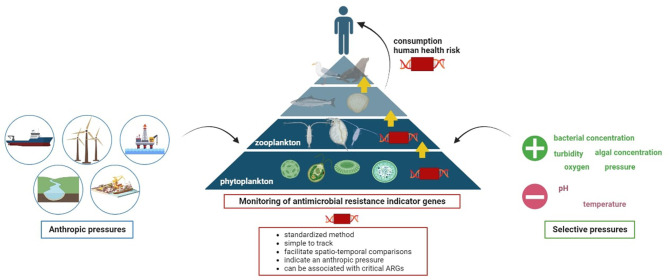

## 1 Introduction

Antimicrobial resistance (AMR) is a major human health concern, involving the transfer of antimicrobial resistant bacteria (ARB) and antimicrobial resistance genes (ARGs) between humans, animals and the environment. In 2019, ~1.27 million deaths were attributed to ARB worldwide (Murray et al., 2022). Without any action to address AMR, this number could increase in the coming years. In fact, the misuse and abuse of antibiotics in human and veterinary medicine as well as in farming have accelerated the emergence of AMR in all environments (Velazquez-Meza et al., 2022). Over the last few years, research on the occurrence of ARGs and ARB in the different environmental compartments has expanded, leading to the publication of comprehensive reviews on AMR factors and pathways in the environment (Singer et al., 2016; Irfan et al., 2022; Larsson and Flach, 2022). These emerging environmental contaminants have been isolated from the soils and sediments (up to 10^9^ gene copies/g), sewage (up to 10^−4^ gene copies/16S rDNA gene copies), rivers (around 10^5^ gene copies/ml), lakes (up to 10^−4^ gene copies/16S rDNA gene copies), seawaters (up to 2.9 log gene copies/ng of DNA) and even in remote areas such as the Arctic (around 10^−4^ gene copies/16S rDNA gene copies) and the clouds (up to 10^4^ gene copies/m^3^ of air; Yang et al., 2017; McKinney et al., 2018; Tan et al., 2018; Nnadozie and Odume, 2019; Alessia et al., 2020; Bourdonnais et al., 2022a; Rossi et al., 2023). Even if the terrestrial and anthropogenic sources are often studied, the role of seas and oceans in the spread of ARGs is still understudied.

It has been estimated that 80% of marine pollution has an anthropogenic origin emanating mainly from direct discharges in the form of effluents from land-bases and from marine activities (fishing activities, passenger and freight traffic) which may be loaded with ARGs and ARB (Amar, 2010). A wide variety of ARGs have been detected in different environmental compartments around the world (Czatzkowska et al., 2022). Targeting all ARGs in the marine environment is time-consuming and costly. Comparing the results from different studies is complex, due to the diversity of methods for detecting and quantifying ARGs, the nature of the environmental samples and the sampling methods. It is necessary to standardize ARG analysis methods and develop indicators of environmental contamination by AMR, which is a well-known issue. These genetic indicators should, by definition, be relatively simple to track in the environment, provide information on global resistance dynamics, indicate anthropogenic pressures, and can be coupled with the search for ARGs to study transfer dynamics (Hocquet et al., 2020). Tracking these indicator genes rather than indicator bacteria allows to compensate for the rapid loss of cultivability of environmental bacteria, and to monitor the large scale spatio-temporal fate of AMR. Some ARGs, frequently occurring in environmental settings that are subjected to human activities, have been proposed as indicator genes to monitor AMR contamination in the environment (Berendonk et al., 2015; Hocquet et al., 2020). By monitoring the *tetA* (tetracycline resistance), *sul1* (sulfonamide resistance) and *intI1* (class 1 integron-integrase) indicator genes in the English Channel and the North Sea seawaters in a previous study, we highlighted the contamination of both coastal and offshore waters by AMR involving potential discharges from land and sea-based activities (Bourdonnais et al., 2022a). In addition to these discharges, marine currents associated with the movements of aquatic organisms will facilitate the long-distance migration of AMR, particularly in plankton communities whose movements are dependent on currents (Hellweger et al., 2016).

Phytoplankton, such as cyanobacteria, diatoms, and cryptophytes, play a crucial role in marine food webs as producers of organic matter and as prey for aquatic herbivores including zooplankton species (Lynam et al., 2017). Most of zooplankton species are filter feeders and are also important in the marine food webs as consumers of phytoplankton and bacterioplankton, and as prey of the upper trophic levels fish. In the English Channel, zooplankton communities are mainly composed of copepods, chaetognaths, and fish larvae (Giraldo et al., 2017). The surface of planktonic cells in the marine environment creates friendly microhabitats for bacteria colonization, especially bacteria of the *Vibrio* genus, through the secretion of substances suitable for their proliferation (Tang et al., 2010). Furthermore, the intestinal mucosa and exoskeleton of some zooplankton species are favorable surfaces for bacterial attachment and development deriving mainly from food materials (Nagasawa and Nemoto, 1988). These bacteria can be potentially pathogenic and their association with plankton can be exacerbated by anthropogenic wastes which transport pollutants into the environment (Hemraj et al., 2017). Bacterial species such as *Escherichia coli, Vibrio alginolyticus, Arcobacter butzleri*, and *Campylobacter lari* have been isolated from small (>64 μm) and large (>200 μm) plankton collected in coastal seawaters near Italy (Maugeri et al., 2004). While some studies have been conducted on the identification of the bacterial flora colonizing planktonic cells (Mestre et al., 2017), their role in the carriage and dissemination of ARGs is still unexplored. However, given that phytoplankton and zooplankton play a fundamental role in aquatic food webs, the presence of ARGs in these organisms could potentially pose a risk of transmission to higher trophic levels, including humans.

To investigate the role of plankton communities (including phytoplankton and zooplankton) in the carriage and transport of AMR, this study focused on the prevalence and abundance of the *tetA, bla*_TEM_ (β-lactam resistance), *sul1*, and *intI1* genes in phytoplankton and zooplankton samples from the English Channel and the North Sea areas. The aim was to determine whether these 4 genes, proposed as indicators for monitoring AMR in the environment, were suitable for such monitoring in these planktonic communities. The total gene abundance was mapped to better assess potential sources of AMR contamination in the environment. However, given the impossibility of establishing a “normal” level of AMR in the samples due to the lack of data, “contamination” levels have been established but do not constitute actual contamination of the samples. Additionally, we assessed the impact of environmental factors and the total bacterial concentration on the abundance of these indicator genes within the phytoplankton and zooplankton communities. Significantly, this study highlighted the role of marine phytoplankton and zooplankton from the English Channel and the North Sea as a reservoir of ARGs, showing their potential involvement in the dissemination of AMR within the marine environment through food webs, potentially affecting human health. This study is an integral part of a research project focusing on the *tetA*, *bla*_TEM_, *sul1*, and *intI1* indicator genes in a benthic food web in the English Channel and the North Sea. The sampling plan was designed considering the position of different marine species in the water column and their marine trophic level. These trophic levels are based on the study conducted by Giraldo et al. (2017) in the English Channel and have been simplified as follows. Level 0 of the food chain corresponds to surface waters, for which results on their “contamination” by the indicator genes have been previously published (Bourdonnais et al., 2022a). The phytoplankton and zooplankton studied in this work belong to levels 1 and 2, respectively. Finally, level 3 consisted of two species of benthic flatfish: *Limanda limanda* (dab) and *Pleuronectes platessa* (plaice; Bourdonnais et al., 2024).

## 2 Materials and methods

### 2.1 Study area

**Figure 1:**
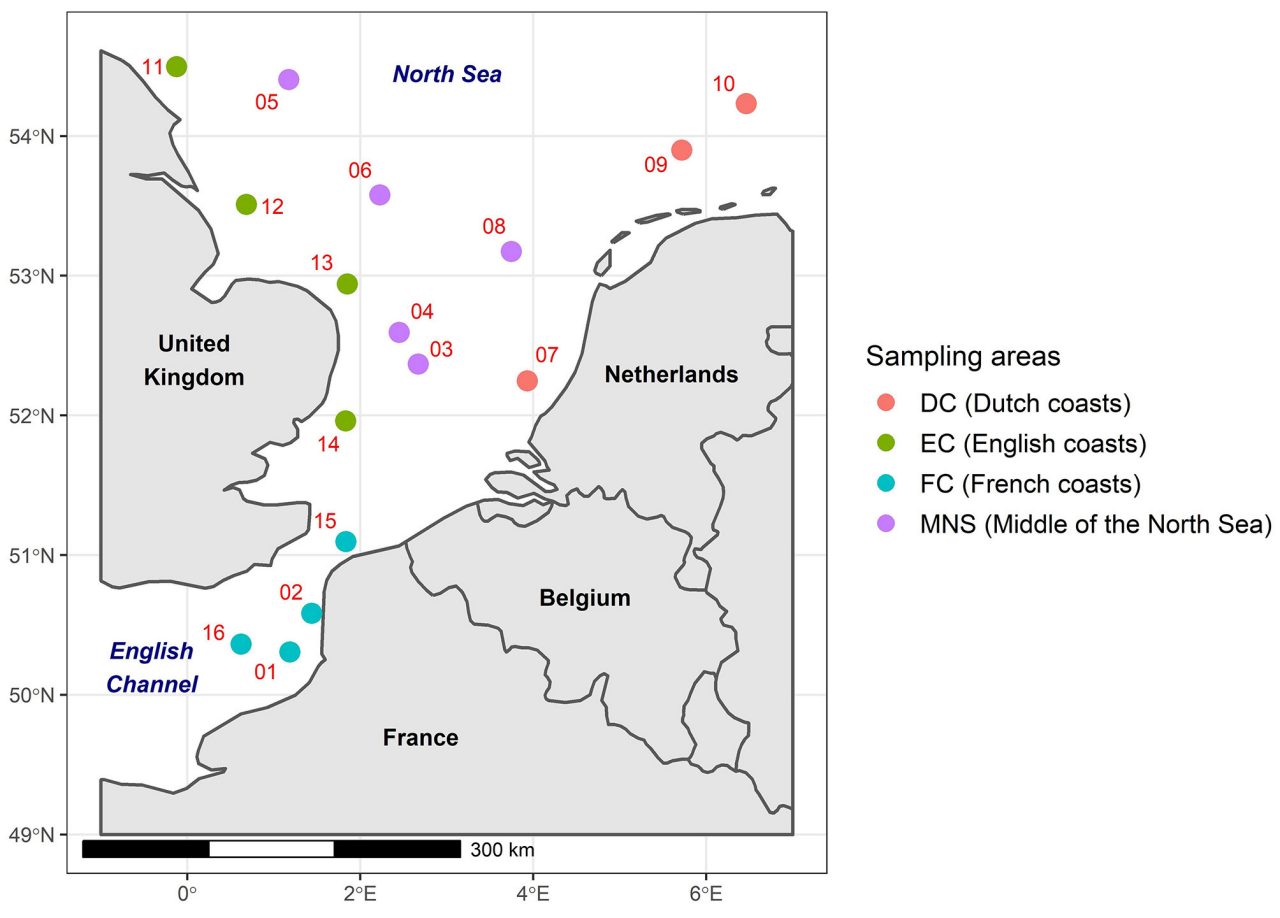
The geographical locations of the phytoplankton and zooplankton sampling sites in the English Channel and the North Sea divided into four defined areas. The red numbers indicate the number of the samples.

The English Channel and the North Sea are located in the northwest of Europe and represent an area of ~650,000 km^2^. These marine regions are significantly influenced by various anthropogenic pressures due to the presence of coastal countries including France, the United Kingdom, Belgium, the Netherlands, Germany, Denmark, and Norway. The English Channel and the North Sea are the receivers of effluents from several rivers crossing these countries that may be contaminated by ARGs, such as the Seine, the Somme (France), the Thames (England), and the Rhine (Netherlands). It is also important to underline the anthropic impacts linked to port activities with the European ports of Le Havre, Rotterdam and Antwerp as well as fishing activities and more globally maritime activities. For the purposes of this study, the English Channel and the North Sea were divided into four distinct areas based on their proximity to the coastal countries. These areas were as follows: the French coasts (FC), the English coasts (EC), the Dutch coasts (DC), and the Middle of the North Sea (MNS; Figure 1).

### 2.2 Sample collection and preparation

The phytoplankton (*n* = 16) and zooplankton (*n* = 16) samples were collected in January 2020 during the IBTS (International Bottom Trawl Survey) oceanographic campaign in the English Channel and the North Sea (Lazard et al., 2021; Figure 1). The phytoplankton samples were collected near the sea surface using a BabyNet with a mesh size of 20 μm attached to a collector. The contents of the collector were then transferred to a 20 μm sieve and rinsed with filtered seawater. For the zooplankton samples, a WP2 net with a mesh size of 200 μm was employed. The contents of the collector were sieved on a 180 μm sieve and then rinsed with filtered seawater. These phytoplankton and zooplankton samples were placed in bottles containing filtered seawater and fixed with 20% (v/v) of glycerol, then stored in a cold room at −20°C on board the vessel before being transferred to the laboratory at −20°C. Protocols of the Ifremer survey are currently being evaluated by the Ifremer and are validated by the ICE IBTS International Group (ICES, 2015). In addition, survey's PIs received training about animal wellbeing and ethics.

At each sampling site, environmental parameters were measured using a Seabird19 bathysonde with different sensors. This probe was immersed near the surface to obtain the following parameters: water temperature (° C), pH, salinity (PSU; Practical Salinity Unit), pressure (mbar), dissolved oxygen (μM/L), turbidity (FTU; Formazin Turbidity Unit) and the concentration of diatoms, cryptophytes, green and blue algae (μg/L). A request for access to these environmental data can be made to the following address: http://data.ifremer.fr/pdmi/portalssearch/main. The distance from the sampling stations to the nearest coastline (km) was estimated.

Before use, each sample was thawed overnight in an oven at 4°C. The samples were centrifuged for 15 min at 4,500 × *g* at 4°C and the pellet was suspended in 10 ml of sterile physiological water. This was performed twice to wash the cells before adding 20% (v/v) of glycerol. All the samples were stored at −20°C until total DNA extraction. Two non-selective enrichments were performed in parallel by diluting the samples at 1/2 with 500 μl of Buffered Peptone Water (BPW, Oxoid, Dardilly, France) and 500 μl of Alkaline Salt Peptone Water (ASPW, Oxoid). These enrichments were incubated for 48 h at 30°C before performing the total DNA extraction. Indeed, these non-selective enrichments in BPW and ASPW were performed to revitalize and multiply the bacteria present in the plankton samples to have a copy number of the four AMR indicator genes above the qPCR limit of quantification, thus making it possible to assess the prevalence of the genes as a complement of their abundance. As there is no specific standard for the preparation of this type of complex environmental sample, we have optimized this enrichment protocol based on three standards: NF EN ISO 4833-1:2013, NF EN ISO 21528-2:2017, and NF EN ISO 21872:2017.

### 2.3 Total DNA extraction

Total DNA was extracted from 1 ml of the samples fixed in glycerol without enrichment and of the BPW/ASPW enrichments with the DNeasy^®^ PowerBiofilm^®^ kit (Qiagen, Hilden, Germany) using the manufacturer's recommendations with some modifications regarding reagent volumes, incubation and vortexing times. The acronyms used in this section refer to the names of the solutions included in the extraction kit. Briefly, all the samples were centrifuged for 5 min at 10,000 × *g*. The pellet was washed with 1 ml of sterile physiological water and centrifuged again. The pellet was then suspended in 400 μl of MBL lysis buffer (instead of 350 μl) and transferred to the bead tubes. One hundred microliters of FB buffer was then added to the tubes before incubating for 5 min at 65°C and shaking for 15 min (instead of 10 min). The tubes were centrifuged for 1 min at 13,000 × *g* and 100 μl of IRS buffer were added to the supernatant and incubated at 4°C for 5 min. After centrifuging the tubes for 1 min at 13,000 × *g*, we added 900 μl of MR buffer and transferred this solution in the centrifugation columns. The tubes were then centrifuged for 1 min at 13,000 × *g*, and the columns were washed with 650 μl of PW buffer and then with 650 μl of absolute ethanol, centrifuging the tubes between each wash step. After drying the columns, DNA was eluted by adding 50 μl of EB buffer twice (instead of 100 μl added once) and its concentration was measured with a DS-11 spectrophotometer (Denovix, Wilmington, United States) at 260 nm. The DNA extracted from the samples without enrichment was then diluted at 1/10 in nuclease-free water before performing the amplification reactions.

### 2.4 Amplification of the AMR indicator genes and the bacterial *tuf* gene by qPCR

The AMR indicator genes *tetA*, *bla*_TEM_, *sul1*, and *intI1* were amplified by qPCR. In order to quantify the total bacterial concentration associated to the phytoplankton and zooplankton samples, we also targeted the bacterial housekeeping *tuf* gene by qPCR, which was originally developed on food samples (Tanaka et al., 2010). As we already optimized the qPCR reaction targeting the *tuf* gene in phytoplankton and zooplankton samples in a previous study, we carried out the experiments once (Bourdonnais et al., 2022b). The primers and probes used for these qPCR reactions are listed in the Table 1. We performed the reactions in a final volume of 20 μl using SYBR Green dye for the qPCR targeting the *tetA*, *bla*_TEM_, and *tuf* genes, and TaqMan probe technology for the qPCR targeting the *sul1* and *intI1* genes. All qPCR reactions were carried out using a LightCycler 480 thermal cycler (Roche, Rotkreuz, Switzerland). Quantification cycle (Cq) values were calculated using the second derivate method. The reagents, amplification conditions and controls used are indicated in our previous publication (Bourdonnais et al., 2022a).

**Table 1:** Primer and probes sequences associated to the qPCR reactions performed in this study.

**Gene**	**Primer/probe sequences**	**qPCR parameters**	**References**
*tuf*	ACHGGHRTHGARATGTTCCG	*E* = 82.97%	Tanaka et al., 2010
	GTTDTCRCCHGGCATNACCAT	*r*^2^ = 0.99	
*tetA*	GCTACATCCTGCTTGCCTTC	*E* = 93.22%	Ng et al., 2001
	CATAGATCGCCGTGAAGAGG	*r*^2^ = 0.99	
*bla*_TEM_	TTCCTGTTTTTGCTCACCCAG	*E* =94.33%	Bibbal et al., 2007
	CTCAAGGATCTTACCGCTGTTG	*r*^2^ = 0.99	
*intI1*	GCCTTGATGTTACCCGAGAG	*E* = 98.34%	Barraud et al., 2010
	GATCGGTCGAATGCGTGT	*r*^2^ = 0.99	
	(6-FAM)ATTCCTGGCCGTGGTTCTGGGTTTT(BHQ1)		
*sul1*	CCGTTGGCCTTCCTGTAAAG	*E* = 109.62%	Heuer and Smalla, 2007
	TTGCCGATCGCGTGAAGT	*r*^2^ = 0.99	
	(FAM)CAGCGAGCCTTGCGGCGG(TAMRA)		

### 2.5 Data analysis and statistics

The prevalence was calculated by dividing the number of samples in which the target gene was detected (with BPW/ASPW enrichment or without enrichment) by the number of samples analyzed, and was expressed as a percentage. The abundance was the concentration of the genes normalized to the concentration of DNA extracted from 1 ml of the phytoplankton or zooplankton samples. We calculated the total abundance of the AMR indicator genes by summing the abundance of each gene for a sample. The total abundance values were classified into four distinct groups to define levels of AMR “contamination” of phytoplankton and zooplankton samples. These levels were: null (no quantification of the indicator genes), low (total abundance below 4 log copies/ng of DNA), moderate (total abundance between 4 and 5 log copies/ng of DNA) and high (total abundance exceeding 5 log copies/ng of DNA). Correlations among the abundance of the AMR indicator genes, the total bacterial concentration and the environmental factors were analyzed using Pearson correlation coefficients (PCC). Only the PCC with a significant *p-*value (assessed by significance test, *p* < 0.05) were considered. We carried out the data presentation and statistical analysis using RStudio Software version 1.4.1717 (RStudio, Inc., Boston, United States) with the “ggplot2” and “corrplot” packages.

## 3 Results

### 3.1 Occurrence of the AMR indicator genes in the phytoplankton and zooplankton communities

**Figure 2:**
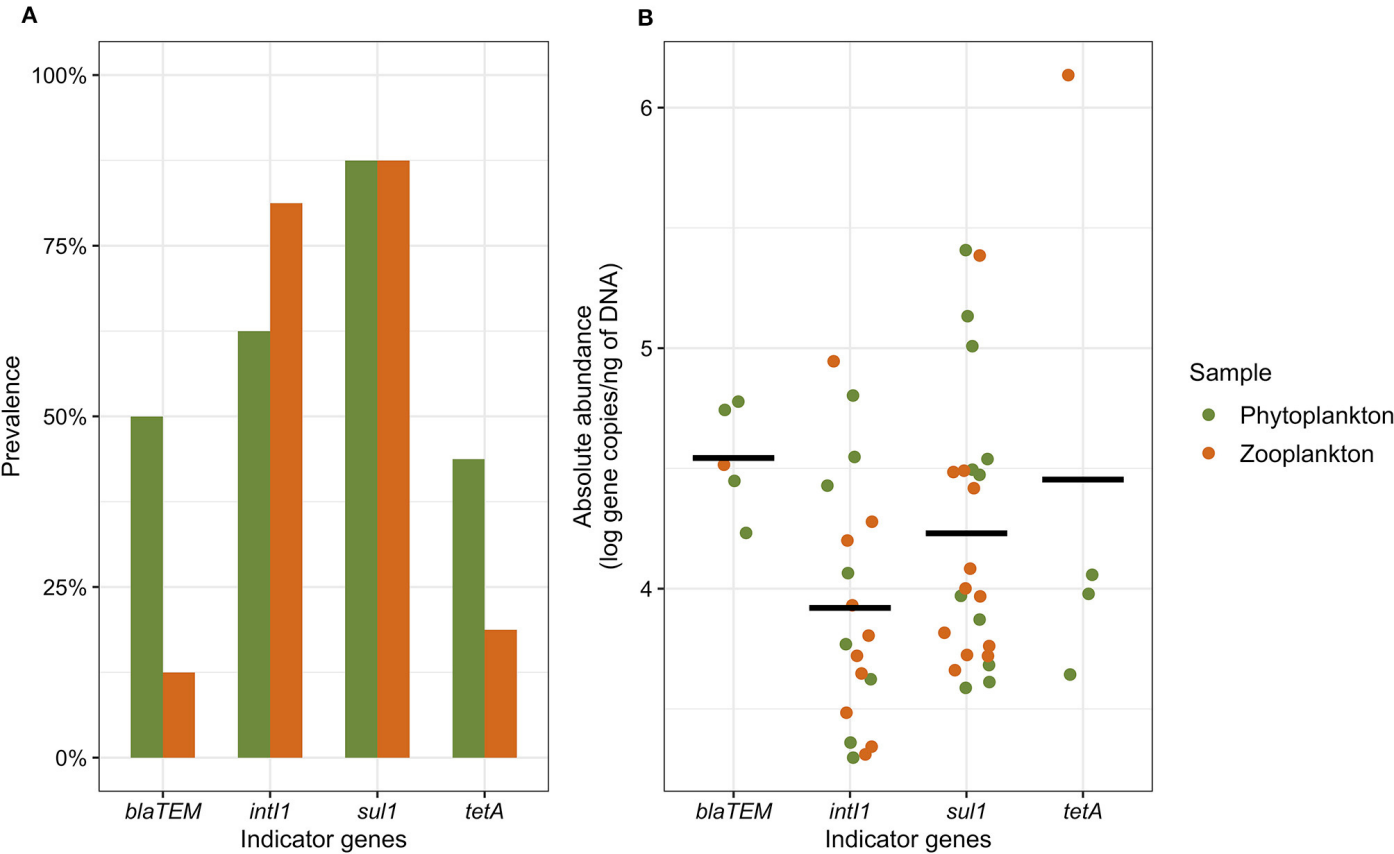
The prevalence **(A)** and abundance **(B)** of the *tetA*, *bla*_TEM_, *sul*, and *intI1* indicator genes in the phytoplankton and zooplankton samples. The prevalence includes the detection of the indicator genes in BPW and ASPW enrichments as well as in raw samples, while the abundance includes the quantification of the indicator genes in the raw samples only. The black bar represents the mean abundance for each gene.

We validated the qPCR results according to the NF T90-471:2015-06 standard (amplification efficiency between 75 and 125%, coefficient of determination *R*^2^ > 0.99 and no quantification of the negative control). All four AMR indicator genes (*tetA*, *bla*_TEM_, *sul1*, and *intI1*) were detected in at least one plankton (phytoplankton and zooplankton) sample. Considering all the plankton communities analyzed in this study (with enrichment in BPW/ASPW and without enrichment; Supplementary Table 1), the prevalence of the AMR indicator genes in the English Channel and the North Sea was found to be 90.6% (93.8% for the phytoplankton samples and 87.5% for the zooplankton samples). Regarding phytoplankton, the *tetA* and *bla*_TEM_ genes were detected in 4/16 BPW enrichments each, but in only 2/16 and 1/16 ASPW enrichments, respectively. The *tetA* gene was detected in two ASPW enrichments performed on zooplankton samples, and the *bla*_TEM_ gene in only one BPW enrichment. In the BPW and ASPW enrichments of the phytoplankton samples, we have more detected the *sul1* gene (11/16 and 9/16, respectively) than the *intI1* gene (4/16 and 7/16, respectively). On the other hand, these two genes were detected as much in BPW enrichments (9/16 samples) as in ASPW enrichments (7/16 samples) for zooplankton. We observed a co-occurrence of the four AMR indicator genes in 21.9% of the plankton samples. The most frequently detected indicator genes in both the phytoplankton and zooplankton communities were *sul1* with a prevalence of 87.5% in both sample types, followed by *intI1*, which exhibited prevalence rates of 62.5 and 81.3% in phytoplankton and zooplankton samples, respectively (Figure 2A). We observed a co-occurrence of these two genes in 71.9% of the phytoplankton and zooplankton samples with mean abundances of 4.2 and 3.9 log copies/ng of DNA, respectively (Figure 2B). In comparison, we observed that the *sul1* gene had a mean abundance of 1.2 log gene copies/ng of DNA in seawater and the *intI1* gene a mean abundance of 1.4 log gene copies/ng of DNA (data not shown; Bourdonnais et al., 2022a). The *bla*_TEM_ gene was detected in half of the phytoplankton samples but in only 12.5% of the zooplankton samples. Similarly, the prevalence of the *tetA* gene was higher in the phytoplankton samples (43.8%) compared to the zooplankton samples (18.8%). The abundance of the *bla*_TEM_ gene ranged from 4.2 to 4.8 log copies/ng of DNA in the plankton samples, while the *tetA* gene exhibited a range of 3.6–4.1 log copies/ng of DNA. Notably, one zooplankton sample exhibited a quantification of 6.1 log copies/ng of DNA for the *tetA* gene. In seawater, the *bla*_TEM_ gene was not quantified and the *tetA* gene was quantified in one sample, at a concentration of 1.6 log gene copies/ng of DNA (Bourdonnais et al., 2022a).

### 3.2 Levels of AMR “contamination” of the phytoplankton and zooplankton communities

**Figure 3:**
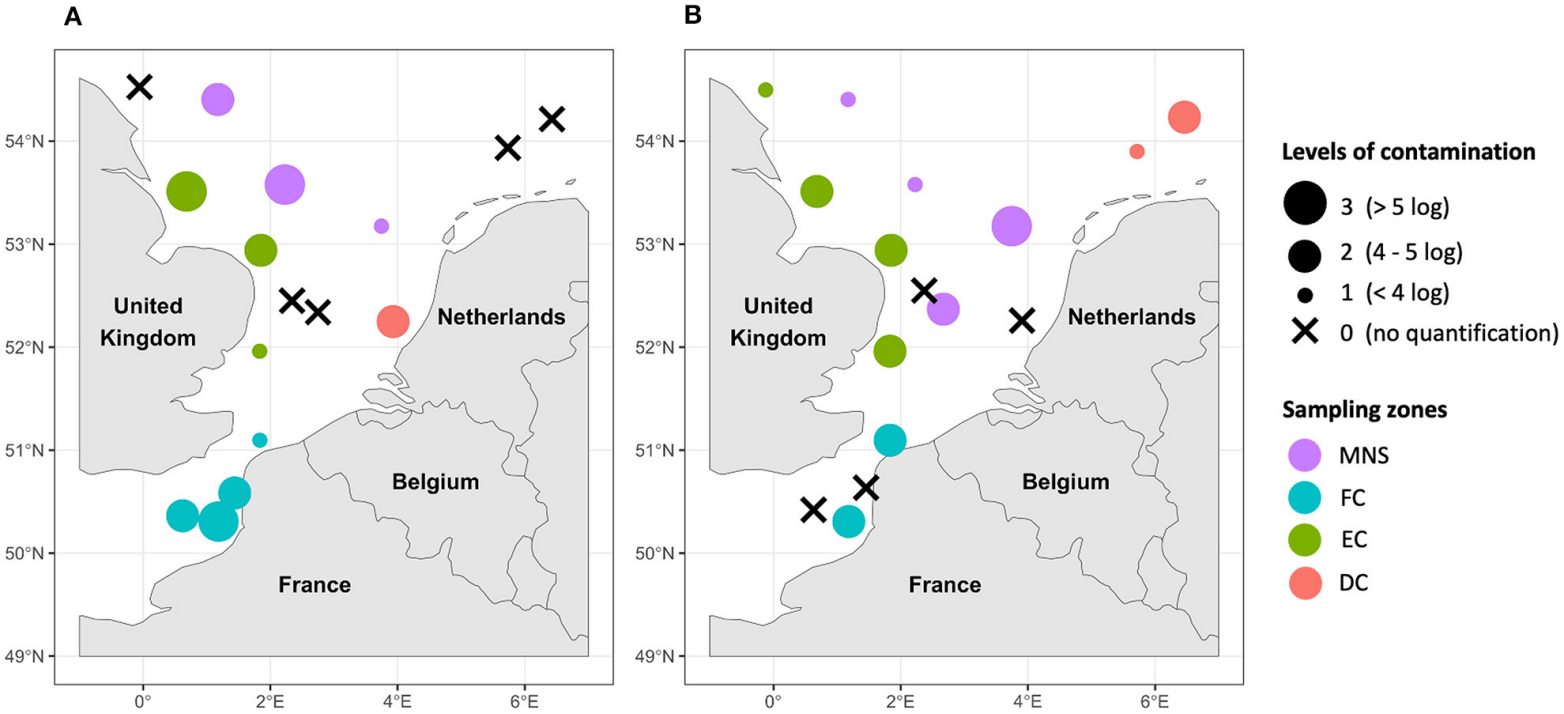
Mapping of the AMR “contamination” levels of the phytoplankton **(A)** and zooplankton **(B)** samples in the English Channel and the North Sea. DC, Dutch coasts; EC, English coasts; FC, French coasts; MNS, Middle of the North Sea. The “contamination” levels correspond to the sum of the abundance of all quantified genes within the same sample (one, two, three, or four genes depending on the gene(s) quantified).

Mappings of AMR “contamination” levels of phytoplankton and zooplankton communities in the English Channel and the North Sea are shown in Figure 3. These “contamination” levels corresponded to the total abundance of the AMR indicator genes which ranged from 0 to 6.2 log copies/ng of plankton DNA. The observed “contamination” levels varied depending on the sampling area and the type of sample analyzed. In the French coastal area, a higher level of “contamination” of phytoplankton communities was detected compared to zooplankton communities except in the North of France near the ports of Dunkirk and Calais. In the same area, up to 2.9 log copies of the *sul1* and *intI1* genes were quantified per 1 ng of DNA in seawater from our previous study (Bourdonnais et al., 2022a). The “contamination” levels of phytoplankton and zooplankton samples varied along the English coasts. Only a high level of AMR “contamination” was noticed in a sample of phytoplankton collected near the Humber (sample n°12), one of the largest estuaries in England, compared to the phytoplankton samples also collected off the English coasts. This level of AMR “contamination” of phytoplankton was associated with co-occurrence of the four indicator genes *tetA*, *bla*_TEM_, *sul1*, and *intI1* with abundances of 4.0, 4.5, 5.0, and 4.4 log gene copies/ng of DNA, respectively (data not shown). The *sul1* and *intI1* genes have also been quantified in this zone between 2 and 3 log gene copies/ng of DNA in seawater (Bourdonnais et al., 2022a). Near the Dutch coasts, the “contamination” level of phytoplankton was higher in the West near Rotterdam than in the North while the opposite trend was observed for zooplankton. The Middle of the North Sea area exhibited a diverse range of “contamination” levels within its phytoplankton and zooplankton communities, which were prominently evident throughout these waters. This could show a significant spread of AMR indicator genes in the North Sea. Notably, one sample of phytoplankton and one sample of zooplankton collected in the Middle of the North Sea exhibited elevated levels of “contamination,” at the same position where we quantified the *tetA, sul1*, and *intI1* genes in seawater (Bourdonnais et al., 2022a).

### 3.3 Correlation between the abundance of the AMR indicator genes, the total bacterial concentration, and the environmental parameters

**Figure 4:**
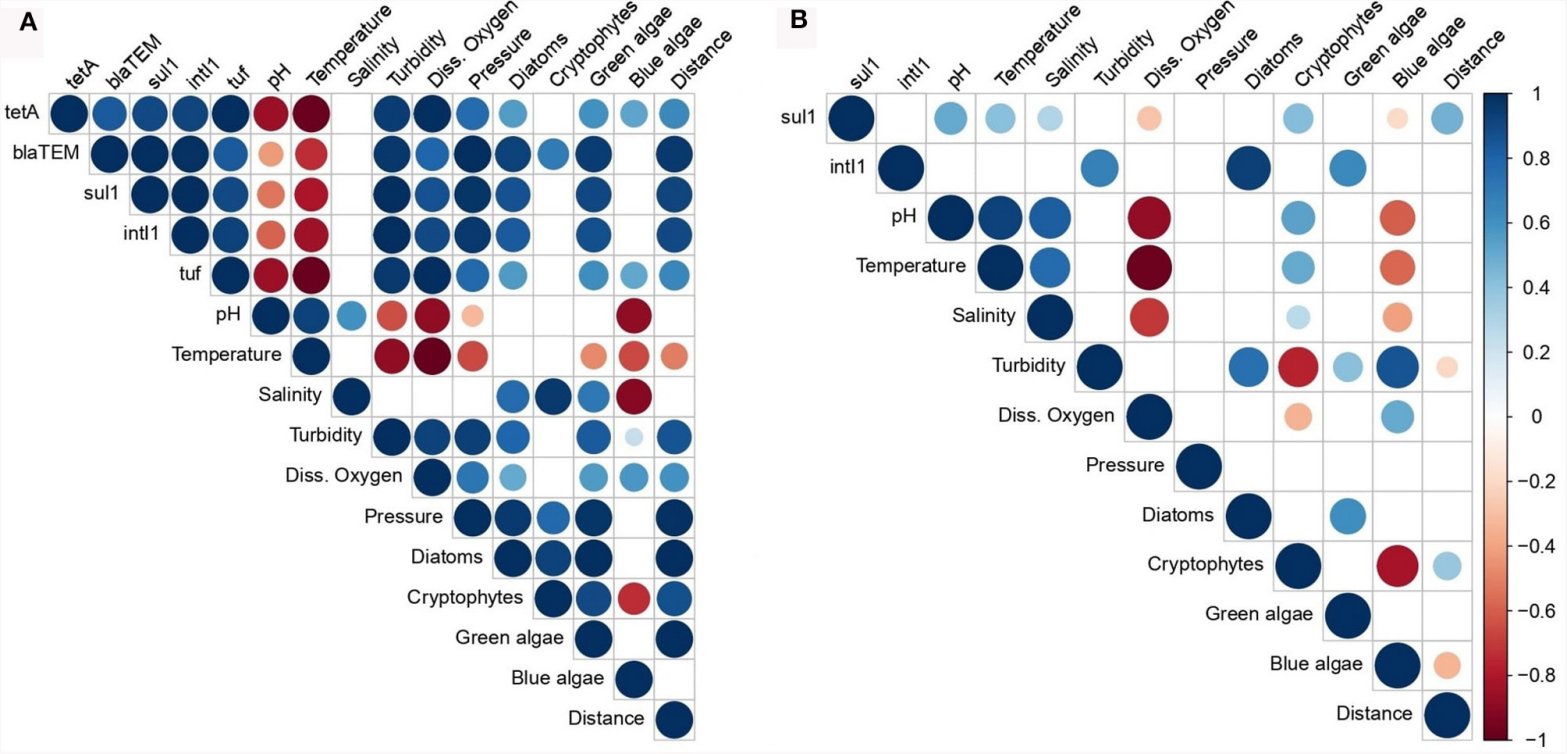
Pearson correlation coefficient (PCC) matrix among the total bacterial concentration, the abundance of the AMR indicator genes and the water parameters associated with the phytoplankton **(A)** and zooplankton **(B)** samples. Diss. oxygen, dissolved oxygen; Distance, distance between the sampling sites and the lands. The color and size keys indicate the PCC.

Pearson correlation coefficient (PCC) was calculated to examine the relationships between the abundance of the AMR indicator genes, the total bacterial concentration (estimated using *tuf* gene quantification) and environmental parameters (Figure 4). In the phytoplankton community, a strong positive correlation was observed among the abundance of the different indicator genes (PCCs > 0.8) with a perfect correlation between the *sul1* and *intI1* genes (PCC = 1.0). Furthermore, the abundance of the *tuf* gene was strongly and positively correlated with the abundance of the *bla*_TEM_, *sul1*, and *intI1* genes and a perfect correlation with the *tetA* gene, suggesting a co-occurrence of indicator genes associated with the bacterial population in the phytoplankton samples. The abundance of the four indicator genes and that of the total bacterial population presented strong positive correlations (PCCs between 0.8 and 1.0) with the measures of water turbidity, dissolved oxygen and pressure values. By contrast, the abundances of all genes were negatively correlated with water pH and temperature. We observed that the abundances of the *bla*_TEM_, *sul1*, and *intI1* indicator genes were correlated with the concentrations of the diatoms and green algae, and the abundances of the *tetA* and *tuf* genes were moderately and positively correlated with the concentrations of these two types of algae. These strong and moderate correlations were noted between the abundance of the same genes and the distance from lands. We did not consider the abundance of the *tetA, bla*_TEM_, and *tuf* genes in zooplankton for the correlation analysis due to their low quantification. Few correlations were significant between the abundance of the AMR indicator genes in the zooplankton samples and the environmental parameters. The abundance of the *sul1* gene was positively and weakly correlated with pH, temperature, salinity values, cryptophytes concentration and the distance from lands with PCCs ranging from 0.3 to 0.5. A stronger positive correlation was observed between the abundance of the *intI1* gene and turbidity, diatoms and green algae concentrations (PCCs between 0.6 and 0.9).

## 4 Discussion

Insufficient attention has been given to the contamination of marine phytoplankton and zooplankton by AMR despite their ecological significance. Phytoplankton, as primary producers, and zooplankton as primary consumers, form the basis of marine food webs. Consumed by the marine species of higher trophic levels, their contamination by AMR represents a risk of accumulation along the food webs up to humans who are final consumers.

In the present study, the monitoring of four indicator genes, including three ARGs and one mobile genetic element, in phytoplankton and zooplankton samples from the English Channel and the North Sea revealed that 90.6% of the plankton communities were carrying at least one AMR indicator gene. The most prevalent and quantified indicators were the *sul1* and *intI1* genes, which encode sulfonamide resistance and class 1 integron-integrase, respectively. The prevalence of the *intI1* gene was higher in zooplankton samples compared to phytoplankton, while the *sul1* gene showed equivalent abundances in both groups, ranging from 3.3 to 6.1 log gene copies/ng of DNA. Moreover, we evidenced a co-occurrence of *sul1* and *intI1* in 71.9% of plankton samples associated with a strong positive correlation between the abundance of these two genes in the phytoplankton samples. These results are consistent with the literature since class 1 integrons are composed, among others, of the *intI1* gene encoding an integrase and the *sul1, sul2*, or *sul3* gene encoding sulfonamide resistance (Stalder et al., 2014). These bacterial genetic elements are capable of acquiring and expressing a large panel of genes as ARGs integrating in cassettes, promoting their dissemination in environment and the emergence of multidrug resistant bacteria (Gillings et al., 2008). Previous studies have highlighted the ubiquity of class 1 integrons in the environment and that their concentration is influenced by anthropogenic pressures (Stalder et al., 2012). The *intI1* gene has actually been suggested as a marker for dissemination of AMR in the environment (Gillings et al., 2015). Indeed, it has been detected in pristine environments, far from human influence and therefore from anthropogenic inputs, such as in sediments in the Golf of Suez and even in sediment, penguin and seal feces from Antarctica, with abundances of up to 4 log gene copies/g (Elsaied et al., 2011; Na et al., 2019), but has not yet been identified associated with marine plankton. The *tetA* and *bla*_TEM_ genes, coding for tetracycline and β-lactam resistance respectively, were the least detected and quantified indicators. These two genes, along with *sul1* and *intI1*, are considered ubiquitous in aquatic environments (Shin et al., 2023). Their low detection in plankton samples compared to *sul1* and *intI1* could be explained by the fact that these two genes are present at a concentration below the limit of quantification of qPCR reactions. Moreover, the *tetA* and *bla*_TEM_ genes were only quantified in phytoplankton and zooplankton samples for which the *sul1* and *intI1* genes had also been quantified. This co-occurrence of genes may be explained by an agglomeration of bacteria with cassettes containing ARGs and located in class 1 integrons, considering the high abundances of the *intI1* gene in these samples. These results are consistent with those obtained in our previous study of seawater contamination in the English Channel and North Sea by the same indicator genes (Bourdonnais et al., 2022a). Indeed, we had demonstrated that the *sul1* and *intI1* genes had prevalences of 42 and 31% in seawater, respectively, while the *bla*_TEM_ gene was not detected and the *tetA* gene was detected in 3% of the seawater samples in the same areas. Moreover, the abundance of the indicator genes was higher in the plankton communities than in seawater. These genes present in seawater can however be assimilated to planktonic organisms present in the marine environment, whether on their surface or even in the gastrointestinal tract of zooplanktonic organisms (Xue et al., 2021). A higher prevalence of these genes was however observed in the phytoplankton samples compared to the zooplankton ones but with similar abundances. Higher abundances of the *sul1* gene than the *tetA* gene were also reported in phytoplankton and zooplankton communities collected along the Ba River in China, up to 10^−3^ gene copies/16S rDNA gene copies (Xue et al., 2021). The authors did not target the *bla*_TEM_ gene in their study but observed low abundances of the *bla*_NDM − 1_, *bla*_IMP − 4_, and *bla*_SHV_ genes also coding for β-lactamases. Similarly, the *intI1* and *sul2* genes were quantified in marine phytoplankton and zooplankton samples between 3.0 × 10^−3^ and 2.9 × 10^−1^ gene copies/16S rDNA gene copies in the Southern North Sea, Irish Sea, and North Atlantic (Di Cesare et al., 2018). In a lake in China, abundances up to 1.0 × 10^6^ copies/g of cells of the *intI1, sul1, sul2, tetA, tetB*, *bla*_TEM_, *qnrB*, and *strA* genes were quantified in cyanobacteria which are a major representative of phytoplankton (Wang et al., 2020). In the light of these prevalence results, we truly believe that the *tetA* and *bla*_TEM_ genes would not be suitable indicators for monitoring AMR contamination of the marine environment unlike the *sul1* and *intI1* genes.

We detected all four *tetA, bla*_TEM_, *sul1*, and *intI1* genes simultaneously in 21.9% of samples, mostly in phytoplankton. The correlation analysis performed in this work confirmed a strong correlation between the abundance of the four indicator genes within the phytoplankton samples. This co-occurrence was associated with high abundances of the indicator genes, up to 6.1 log gene copies/ng of plankton DNA, and therefore with the presence of potential sources of contamination at the sample collection areas. We defined “contamination” levels of the samples based on the total abundance of the indicator genes and mapped them. Thus, we observed disparities in the “contamination” of plankton samples depending on the sampling area. High “contamination” of plankton by AMR has been reported near the French and English coasts. Contamination of surface seawater and flatfish samples in these areas with the *sul1* and *intI1* genes was reported in previous studies and could reveal multiple sources of contamination (Bourdonnais et al., 2022a, 2024). Indeed, French coastal waters are subject to river effluents such as the Somme, at the mouth of which we observed significant “contamination” of phytoplankton and zooplankton samples by AMR. There is a worldwide consensus that even after their treatment, wastewater remain loaded with ARGs and are discharged into rivers which provide a reservoir of ARGs that can be exchanged between environmental bacteria before being transported to the marine environment (Cacace et al., 2019). In addition, there are differences in wastewater treatment regulations between the European countries which can lead to greater or lesser discharges in the sea. Contamination of plankton samples has also been observed near the English coast, not far from the mouth of the Thames and the Humber estuary. The presence of *V. parahaemolyticus* strains phenotypically resistant to kanamycin, gentamicin, cefazolin, and tetracycline has previously been reported in the Humber estuary which is the largest source of water discharged from England into the North Sea (Daramola et al., 2009). As well, contamination of the Thames with sulfonamide antibiotics and high abundances of the *ermB*, *bla*_TEM_, *tetA, tetG, tetW, sul1, sul2, intI1*, and *intI2* genes have been relayed in several studies (White et al., 2019; Xu et al., 2019). We also quantified the AMR indicator genes in the Middle of the North Sea, an area theoretically subject to little anthropogenic impact, due to its remote distance from the coast. The abundance of the indicator genes in the phytoplankton samples was positively correlated with distance from land demonstrating that AMR in the marine environment is not only affected by terrestrial sources. Indeed, in a previous study, the authors also highlighted a correlation between distance from land and the total abundance of ARGs such as *tetA, tetB, sul1, ermB*, and *bla*_TEM_ in the Southern Ocean in Antarctic (Jang et al., 2022). The occurrence of AMR in planktonic communities in the North Sea could therefore have several origins. The presence of offshore platforms (windfarms, oil, and gas platforms) in this area could play a role in the spread of ARGs by the various effluents or marine animals such as birds, fish and mammals carrying AMR that may be attracted by these platforms (Vanermen et al., 2015). This dissemination of AMR indicators in the open sea raises questions about the involvement of maritime activities and the various discharges that this implies such as wastewater and ballast water that is pumped and discharged into the sea. A recent study revealed a high diversity of ARGs in ballast water from ships coming from all over the world (Australia, Japan, Europe, Korea, and South America) that docked in two Chinese ports (Lv et al., 2020). For example, the *sul1, sul2, tetM, tetQ, ermB, strB*, and *intI1* genes were quantified in these waters with abundances up to 6 log copies/ml, providing evidence that ballast waters are a reservoir of ARGs but also an important factor of their dissemination in the marine environment. Moreover, ocean currents are also a source of ARG transport over very long distances on a global scale (Zhang et al., 2022).

The occurrence of ARGs in plankton communities and their dispersion in the marine environment is mainly linked to the presence of bacteria. Indeed, planktonic cells are associated with a rich and diverse bacterial flora, mainly Cyanobacteria, Proteobacteria, Bacteroidetes, and Actinobacteria, involved in the carriage and transfer of ARGs (Zhao et al., 2022; Wang et al., 2023). Indeed, free bacteria in the water column are sensitive to environmental stresses such as predation, viral lysis and physicochemical conditions of the marine environment. Planktonic cells may provide a refuge for bacteria against these external hazards, mainly protozooplankton that may harbor endosymbiotic bacteria, such as *Legionella pneumophila* and *Vibrio cholerae*, capable of surviving the action of disinfectants that normally kill free-living bacteria (Tang et al., 2010). To assess the role of bacteria population in the carriage of AMR indicator genes among plankton communities, we quantified the bacterial housekeeping *tuf* gene in the phytoplankton and zooplankton samples. This gene was quantified in all phytoplankton samples, its abundance being positively correlated with all AMR indicator genes. This shows that phytoplankton-associated bacteria are potential hosts of the *tetA*, *bla*_TEM_, *sul1*, and *intI1* genes. On the other hand, the *tuf* gene was poorly quantified in zooplankton samples. This could be explained by the fact that the totality of the bacterial flora associated with these samples was not targeted by the *tuf* gene and/or that the quantified genes were in the form of free DNA. It was found that a very low density of bacteria was associated with copepods, the main representatives of zooplankton, making their quantification difficult by molecular biological methods (Brandt et al., 2010). The low quantification of the *tuf* gene could come from a heterogeneity of colonization of zooplankton by bacteria given the diversity of species composing this zooplankton such as larvae of vertebrates, invertebrates, krill and copepods. These species have different immune systems and can therefore be colonized by a variety of bacterial species depending on the environment where they live. In a recent study, *Aeromonas* strains isolated from marine copepods in the Seine estuary had phenotypic resistances to sulfamethoxazole, ertapenem, and some penicillins and cephalosporins (Chaix et al., 2017). Some isolates even had resistance to up to 6 antibiotics indicating that zooplankton can be an important vector of ARGs-carrying bacteria. Moreover, an *in vivo* study carried out on zooplankton species, *Daphnia* spp., demonstrated that the gastrointestinal tract of these crustaceans was suitable to the transfer of the *vanA* plasmid gene coding for vancomycin resistance between *Enterococcus faecalis* strains, which could be spread in the environment through fecal deposition (Olanrewaju et al., 2019). The quantification of AMR indicator genes could be relicate extracellular DNA, or be associated to bacteriophages, an important vector in the dissemination of AMR. Bacteriophages are the most abundant biological entities in the marine environment. It has been estimated that the number of bacteriophages may be 10 times greater than the number of bacteria (Wommack and Colwell, 2000). In a study carried out in the Mediterranean Sea, a high abundance of the *bla*_TEM_, *bla*_CTX − M−1_, *bla*_CTX − M−9_, *sul1*, and *tetW* genes was observed in bacteriophage particles, ranging from 4 to 9 log gene copies/L of filtered water (Blanco-Picazo et al., 2020).

In order to evaluate the environmental factors influencing the occurrence, the persistence and the dissemination of the indicator genes into planktonic communities, a correlation analysis was carried out. The abundance of the *tetA*, *bla*_TEM_, *sul1*, and *intI1* genes in phytoplankton were positively correlated with turbidity and dissolved oxygen which was consistent with the results from our previous study involving the same indicator genes quantified in seawater (Bourdonnais et al., 2022a). The turbidity measurement near the surface may reflect the presence of suspended matter such as microorganisms but also phytoplankton and zooplankton on which microorganisms can aggregate. A positive correlation was observed between turbidity, diatom and green algae concentration, and the *tuf* gene abundance expressing the total bacterial concentration. Turbidity can also indicate abiotic materials such as microplastics that are known to be hotspots for ARGs carried by ARB. These ARB aggregate on these particles and can form biofilms, thus promoting the transfer of ARGs between bacteria and their long-distance transport in marine environment (Liu et al., 2021). High turbidity, expressed by high concentration of phytoplankton cells, would cause an increase in the photosynthetic process in addition to a high amount of microorganisms associated with phytoplankton. This could explain the strong positive correlation between the *tuf* gene abundance, turbidity, dissolved oxygen and the algae concentration such as diatoms and green algae. Sampling phytoplankton at different times of the day/night could confirm the involvement of photosynthesis in maintaining the AMR associated with these microorganisms. Moreover, turbid waters would limit the penetration of UV rays thus protecting microorganisms and ARGs from degradation. Similar observations were reported in river waters in Germany where the abundance of the *bla*_TEM_, *sul1, tetM*, and *ermB* resistance genes was strongly and positively correlated with turbidity (Reichert et al., 2021). As well, higher water turbidity in an estuary in China was associated with high abundances of the *sul2, sul3, tetW*, and *intI1* genes, implying that suspended particulate matter could be a reservoir of ARGs (Chen et al., 2020). Few studies have focused on the effect of changes in dissolved oxygen concentration on the occurrence of ARGs in marine environment. High concentrations of dissolved oxygen would therefore be an environmental factor promoting the growth of certain bacteria potentially carrying ARGs, such as *Xanthomonas, Bacteroides, Lactobacillus*, and *Porphyromonas* in wastewater treatment systems (Tao et al., 2016). An *in vitro* study showed that the maintenance of *E. coli* strains carrying the *tetC* gene on a plasmid was favored under aerobic rather than anaerobic conditions (Rysz et al., 2013). These results suggest that biotic and abiotic factors related to the marine environment are likely to influence the occurrence of ARGs and MGE among plankton communities. In a recent study, the authors highlighted direct and indirect effects of phytoplankton and zooplankton communities in the dynamics of AMR (Xue et al., 2021). The direct effect is characterized by the secretion of organic carbon by these plankton communities that protect ARGs from degradation by nucleases present in the environment. Concerning the indirect effect, it has been demonstrated that organic matter produced by plankton is a source of nutrients for bacteria, the main hosts of ARGs, which would then come to fix themselves on the surface of the planktonic cells, hence posing a great threat to public health. Considering the central position of plankton in marine food webs, there is a risk of AMR spreading to the upper levels of trophic chains, which can be assimilated to the phenomenon of bioaccumulation that has already been demonstrated with antibiotic molecules in both terrestrial and aquatic food chains (Hu et al., 2023). The presence of the *tetA*, *bla*_TEM_, *sul1*, and *intI1* AMR indicator genes was in fact observed on the skin, gills and intestine of wild flatfish from the English Channel and the North Sea (*Limanda limanda* and *Pleuronectes platessa*), marine species destined for human consumption and constituting the last trophic level (Bourdonnais et al., 2022). Abundances were as high as 5.6 log gene copies/ng of DNA, higher than in phytoplankton and zooplankton communities. In order to monitor AMR in the marine environment by implementing indicator genes, further studies need to be conducted to accumulate more data and thus define the “normal” background levels of AMR in marine environments (Bengtsson-Palme et al., 2023). This will enable us to identify the areas where AMR exceeds the “normal” level, i.e., the areas that may warrant further investigation.

## 5 Conclusion

This study shed new light on the role of phytoplankton and zooplankton communities in the English Channel and North Sea in the spread of ARGs. The tracking of the *tetA*, *bla*_TEM_, *sul1*, and *intI1* genes, proposed as indicators of environmental contamination by AMR, revealed that marine phytoplankton and zooplankton are significant vectors for the transport of AMR. These indicator genes had an overall prevalence of 90.6% in the plankton communities from the English Channel and the North Sea. The *sul1* and *intI1* genes were detected in more than 60% of the samples, with mean abundances of 4.2 and 3.9 log gene copies/ng of DNA, respectively. This monitoring has also highlighted that there are different potential sources of AMR contamination in the English Channel and the North Sea, notably from land-based sources near the French and English coasts. There is also a significant spread in the marine environment until the open sea favored by marine currents. The long-range dispersal of ARGs by phytoplankton and zooplankton represents a critical pathway for the extensive dissemination of these genes, posing a significant threat to human health. Further studies are needed to monitor the *sul1* and *intI1* genes, which would be good indicators of AMR contamination of the environment unlike the *tetA* and *bla*_TEM_ genes, in order to gain a better understanding of the risk to human health posed by AMR contamination of plankton communities and marine food webs in general. Indeed, we highlighted in our large-scale project that seawater, plankton and flatfish constituting a benthic food web played a crucial role not only in marine ecosystems but also in the dissemination of AMR.

## Data availability statement

The original contributions presented in the study are included in the article/Supplementary material, further inquiries can be directed to the corresponding author.

## Ethics statement

The manuscript presents research on animals that do not require ethical approval for their study.

## Author contributions

EB: Conceptualization, Data curation, Formal analysis, Investigation, Methodology, Writing – original draft, Writing – review & editing. CL: Supervision, Validation, Writing – review & editing. TB: Conceptualization, Supervision, Validation, Writing – review & editing. GM: Conceptualization, Supervision, Validation, Writing – review & editing.

## Supplementary material

Supplementary Table 1 Detection of the *tetA*, *bla*_TEM_, *sul1*, and *intI1* indicator genes in the phytoplankton (PH) and zooplankton (ZO) samples by qPCR. (+), Detection; (-), No Detection; BPW, Buffered peptone water enrichment; ASPW, Alkaline salt peptone water enrichment; No enrich., No enrichment.
