# Supplementary material for: Monitoring indicator genes to assess antimicrobial resistance contamination in phytoplankton and zooplankton communities from the English Channel and the North Sea
Source: Front Microbiol. 2024 Feb 8;15:1313056. doi: 10.3389/fmicb.2024.1313056 (PMC10882542; doi:10.3389/fmicb.2024.1313056)
Supplement: Supplementary Table 1 — Detection of the tetA, blaTEM, sul1, and intI1 indicator genes in the phytoplankton (PH) and zooplankton (ZO) samples by qPCR. (+), Detection; (-), No Detection; BPW, Buffered peptone water enrichment; ASPW, Alkaline salt peptone water enrichment; No enrich., No enrichment. [file Table_1.docx]

|  | ***tetA*** | | | ***bla*_TEM_** | | | ***sul1*** | | | ***intI1*** | | |
| --- | --- | --- | --- | --- | --- | --- | --- | --- | --- | --- | --- | --- |
| Sample | BPW | ASPW | No enrich. | BPW | ASPW | No enrich. | BPW | ASPW | No enrich. | BPW | ASPW | No enrich. |
| PH01 | - | - | **+** | - | - | **+** | **+** | **+** | **+** | **+** | **+** | **+** |
| PH02 | - | - | - | - | - | **+** | **+** | - | **+** | - | - | - |
| PH03 | **+** | - | - | **+** | - | - | **+** | - | - | **+** | **+** | - |
| PH04 | - | - | - | - | - | - | - | - | - | - | - | - |
| PH05 | - | - | - | - | - | - | **+** | - | **+** | - | - | **+** |
| PH06 | - | - | **+** | - | - | **+** | - | **+** | **+** | - | - | **+** |
| PH07 | - | - | - | - | - | - | - | **+** | **+** | - | **+** | **+** |
| PH08 | - | - | - | - | - | - | **+** | - | **+** | - | - | - |
| PH09 | - | - | - | **+** | - | - | - | - | - | - | - | - |
| PH10 | - | - | - | **+** | - | - | **+** | **+** | - | **+** | **+** | - |
| PH11 | **+** | - | - | - | - | - | **+** | **+** | - | - | - | - |
| PH12 | **+** | **+** | **+** | - | **+** | **+** | **+** | **+** | **+** | - | **+** | **+** |
| PH13 | - | **+** | - | **+** | - | - | **+** | **+** | **+** | **+** | **+** | **+** |
| PH14 | - | - | - | - | - | - | **+** | - | **+** | - | - | **+** |
| PH15 | **+** | - | - | - | - | - | **+** | **+** | **+** | - | - | - |
| PH16 | - | - | - | - | - | - | - | **+** | **+** | - | **+** | **+** |
| Prevalence | 43.75% | | | 50.00% | | | 87.50% | | | 62.50% | | |
| ZO01 | - | - | - | - | - | - | **+** | **+** | **+** | **+** | **+** | **+** |
| ZO02 | - | - | - | - | - | - | **+** | **+** | - | **+** | - | - |
| ZO03 | - | - | - | - | - | - | - | **+** | **+** | - | **+** | **+** |
| ZO04 | - | - | - | - | - | - | - | **+** | - | - | **+** | - |
| ZO05 | - | **+** | - | - | - | - | **+** | **+** | **+** | **+** | - | **+** |
| ZO06 | - | - | - | - | - | - | **+** | - | **+** | - | - | - |
| ZO07 | - | - | - | - | - | - | - | - | - | - | - | - |
| ZO08 | - | - | **+** | - | - | **+** | - | - | **+** | - | - | **+** |
| ZO09 | - | **+** | - | **+** | - | - | **+** | **+** | **+** | **+** | **+** | - |
| ZO10 | - | - | - | - | - | - | - | - | **+** | - | - | **+** |
| ZO11 | - | - | - | - | - | - | **+** | - | **+** | **+** | - | **+** |
| ZO12 | - | - | - | - | - | - | **+** | **+** | **+** | **+** | **+** | **+** |
| ZO13 | - | - | - | - | - | - | - | - | **+** | **+** | **+** | **+** |
| ZO14 | - | - | - | - | - | - | **+** | - | **+** | **+** | **+** | **+** |
| ZO15 | - | - | - | - | - | - | **+** | - | **+** | **+** | - | **+** |
| ZO16 | - | - | - | - | - | - | - | - | - | - | - | - |
| Prevalence | 18.75% | | | 12.50% | | | 87.50% | | | 81.25% | | |
